# Avoidance prone individuals self reporting behavioral inhibition exhibit facilitated acquisition and altered extinction of conditioned eyeblinks with partial reinforcement schedules

**DOI:** 10.3389/fnbeh.2014.00347

**Published:** 2014-10-06

**Authors:** Michael Todd Allen, Catherine E. Myers, Richard J. Servatius

**Affiliations:** ^1^School of Psychological Sciences, University of Northern ColoradoGreeley, CO, USA; ^2^Stress and Motivated Behavior Institute, NJMS-UMDNJNewark, NJ, USA; ^3^Neurobehavioral Research Lab, DVA Medical Center, NJHCSEast Orange, NJ, USA

**Keywords:** behavioral inhibition, partial reinforcement, eyeblink conditioning, associative learning, anxiety disorders

## Abstract

Avoidance in the face of novel situations or uncertainty is a prime feature of behavioral inhibition which has been put forth as a risk factor for the development of anxiety disorders. Recent work has found that behaviorally inhibited (BI) individuals acquire conditioned eyeblinks faster than non-inhibited (NI) individuals in omission and yoked paradigms in which the predictive relationship between the conditioned stimulus (CS) and unconditional stimulus (US) is less than optimal as compared to standard training with CS-US paired trials (Holloway et al., 2014). In the current study, we tested explicitly partial schedules in which half the trials were CS alone or US alone trials in addition to the standard CS-US paired trials. One hundred and forty nine college-aged undergraduates participated in the study. All participants completed the Adult Measure of Behavioral Inhibition (i.e., AMBI) which was used to group participants as BI and NI. Eyeblink conditioning consisted of three US alone trials, 60 acquisition trials, and 20 CS-alone extinction trials presented in one session. Conditioning stimuli were a 500 ms tone CS and a 50-ms air puff US. Behaviorally inhibited individuals receiving 50% partial reinforcement with CS alone or US alone trials produced facilitated acquisition as compared to NI individuals. A partial reinforcement extinction effect (PREE) was evident with CS alone trials in BI but not NI individuals. These current findings indicate that avoidance prone individuals self-reporting behavioral inhibition over-learn an association and are slow to extinguish conditioned responses (CRs) when there is some level of uncertainty between paired trials and CS or US alone presentations.

## Introduction

Anxiety disorders are the most common form of mental illness. However, the development of anxiety disorders is unclear. Two individuals can experience the same event and yet one develops an anxiety disorder while the other does not. Many factors including genetics, gender, personality and prior experiences are hypothesized to play a role in the development of anxiety disorders (Mineka and Zinbarg, 2006). Recent work has focused on a learning diathesis model that involves differences in learning based upon specific temperament factors such as behavioral inhibition or BI.

Behavioral inhibition has been put forth as a possible risk factor for the development of anxiety disorders (Fox et al., 2005). Behavioral inhibition is defined as a temperamental tendency to withdraw from or avoid novel social and non-social situations (Kagan et al., 1987; Morgan, 2006). Another feature of BI is a sensitivity to forming associations between stimuli. Recent studies examining classical conditioning with individuals expressing behavioral inhibition have found enhanced acquisition of conditioned eyeblinks (Myers et al., 2011; Caulfield et al., 2013; Holloway et al., 2014). Classically conditioned eyeblink conditioning involves the pairing of a conditioned stimulus (CS) tone with an unconditional stimulus (US) corneal air puff which result in learning a conditioned response (CR) eyeblink to the previously neutral CS. There is also a long history indicating that classical conditioning of the eyeblink or eyelid response is affected by anxiety (Hilgard et al., 1951; Spence and Taylor, 1951; Taylor, 1951; Spence and Farber, 1953; Baron and Connor, 1960; King et al., 1961; Beck, 1963; Spence et al., 1964; Spence and Spence, 1966). Consistent with recent findings with BI, these studies revealed enhanced CR acquisition including greater asymptotic performance and a greater number of CRs overall compared to individuals reporting low anxiety.

In addition to a long history of behavioral work in humans and animals, the neural substrates of classical eyeblink conditioning are well understood. Cerebellar and brainstem circuits are known to underlie acquisition, retention, and extinction of eyeblink conditioning across several mammalian species including rabbits, rodents, and humans (for review see Thompson and Steinmetz, 2009). The present study utilized delay conditioning in which the CS and US partially overlap and co-terminate. This form of eyeblink conditioning is known to require the cerebellum, but not other brain structures such as the hippocampus (Schmaltz and Theios, 1972; Gabrieli et al., 1995) or cerebral cortex (Mauk and Thompson, 1987). However, strong evidence also exists for the associative learning in the cerebellum during delay conditioning to be modified by septo-hippocampal (Berry and Thompson, 1979; Allen et al., 2002) and amygdala inputs (Whalen and Kapp, 1991; Weisz et al., 1992; Blankenship et al., 2005). Stein et al. (2007) found that anxiety prone subjects enhanced exhibited amygdala activity during the processing of emotional stimuli. If anxiety vulnerable individuals have greater amygdala activity than non-vulnerable individuals in response to the mildly aversive corneal air puff, this activity could facilitate associative learning in the cerebellum for eyeblink conditioning. These limbic systems may be one mechanism through which temperamental factors such as behavioral inhibition facilitate acquisition of classically conditioned eyeblinks.

Another possible explanation for enhanced acquisition of eyeblink CRs in behaviorally inhibited (BI) individuals is an avoidance of the US air puff by eye closure in response to the CS tone. Holloway et al. (2014) tested the possibility of enhanced avoidance learning in BI individuals using a delay, omission, or yoked conditioning schedule. Omission training was identical to delay, except that the performance of a CR by the participant resulted in omission of the US on that trial. Avoidance learning in eyeblink conditioning has been defined as the degree to which learning during omission training exceeds that of the yoked controls (Logan, 1951; Gormezano et al., 1962; Moore and Gormezano, 1963). Holloway et al. (2014) failed to observe avoidance learning in BI individuals, but did observe enhanced acquisition relative to non-inhibited (NI) individuals. The greater facilitation of learning in the omission and yoked groups was evident in situations of partial reinforcement due to the omission of the US on some trials. These findings were interpreted as an increased sensitivity to uncertainty in BI individuals in the case of partial reinforcement.

In addition to avoidance, BI also includes social reticence and enhanced reactivity to novelty, threat, and uncertainty (Hirshfeld et al., 1992; Schwartz et al., 2003a,b). Grupe and Nitschke (2013) defined anxiety as “anticipatory affective, cognitive, and behavioral changes in response to uncertainty about a potential future threat” (p.489). Anxiety disorders may come about due to how an individual learns to respond to environmental cues, especially when there is some uncertainty about relationships between stimuli. Examples of uncertainty in classical conditioning would include schedules of partial reinforcement.

Partial reinforcement for classical eyeblink conditioning has been defined by Leonard and Theios (1967) based on the US air puff being the reinforcing event. Therefore, partial reinforcement in eyeblink conditioning involves CS tone alone trials that omit the US air puff. Various manipulations of schedules of partial reinforcement involving CS alone and CS-US paired trials in human eyeblink conditioning have produced three major findings. These results include a significant decrement in acquisition in the partial reinforcement group as compared to the continuous reinforcement group (Reynolds, 1958; Ross, 1959; Hartman and Grant, 1960; Ross and Spence, 1960; Runquist, 1963; Perry and Moore, 1965), a partial reinforcement extinction effect (PREE; Longenecker et al., 1952; Perry and Moore, 1965; Newman, 1967; Leonard, 1975), and a null effect of no significant differences in acquisition between partial and continuous reinforcement schedules (Humphreys, 1939; Grant et al., 1950; Hake and Grant, 1951; Grant and Schipper, 1952; Moore and Gormezano, 1963; Price et al., 1965; Foth and Runquist, 1970).

In the current study, we investigated the effects of BI on two forms of partial reinforcement. Based on the levels of responding in omission and yoked groups in the Holloway et al. (2014) study, we chose a 50% partial reinforcement schedule with CS alone trials intermixed with CS-US paired trials. This schedule is also the most common partial reinforcement schedule from the human eyeblink conditioning literature. In addition, we included a 50% partial US group to test the effects of US alone rather than CS alone trials inter-mixed with CS-US paired trials. The inclusion of un-signaled air puff USs would be a different type of unexpected trial type.

Based on the omission and yoked results of Holloway et al. (2014), we hypothesized that 50% partial reinforcement (either with CS alone or US alone trials) would result in reduced conditioned responding as compared to 100% paired trials. In addition, we hypothesized there would be enhanced acquisition of CRs in BI individuals as compared to NI individuals. We also hypothesized that partial reinforcement with CS alone trials would result in a PREE based on previous eyeblink conditioning experiments schedule (Longenecker et al., 1952; Perry and Moore, 1965; Newman, 1967; Leonard, 1975).

## Materials and methods

### Participants

One hundred forty nine college-aged students were recruited from the University of Northern Colorado, School of Psychology. Students voluntarily participated to receive class credit or extra credit for psychology classes. Ninety eight females and 51 males with mean age of 19.9 (SD = 3.0, range 18–38) and mean education of 13.5 years (SD = 1.4, range 11–19) were included in the study. Informed consent was obtained in accordance with procedures approved by the University of Northern Colorado Institutional Review Board adhering to the federal regulations on research involving human subjects.

### Materials and apparatus

The eyeblink conditioning apparatus and procedures were similar to that previously described (Beck et al., 2008). The tone stimulus was produced with Coulbourn Instruments (Allentown, PA, USA) signal generators and passed to a David Clark aviation headset (Model H10–50, Worchester, MA, USA). Sound levels were verified with a Realistic sound meter (RadioShack, Fort Worth, TX, USA). The headset was fitted with a boom placed 1 cm from the cornea that delivered a 5 psi air puff US via sylastic tubing connected to a regulator and released by a computer controlled solenoid valve (Clipper Instruments, Cincinnati, OH). To record the eyelid electromyographic (EMG) signal, pediatric silver/silver chloride EMG electrodes with solid gel were placed above and below the left eye, with the ground electrode placed on the neck. The EMG signal was passed to a medically isolated physiological amplifier (UFI, Morro Bay, CA, USA), low-pass filtered and amplified 10 K. The EMG signal was sampled at 500 Hz by an A/D board (PCI 6025E, National Instruments, Austin, TX, USA) connected to an IBM-compatible computer. Software control of stimulus generation was performed by LabView (National Instruments).

### Psychometric scales

Study participants completed the Adult Measure of Behavioral Inhibition or AMBI (Gladstone and Parker, 2005). The AMBI is a 16-item self-report inventory that assesses current tendency to respond to new stimuli with inhibition and/or avoidance, and has also been shown to be a measure of anxiety proneness.

### BI groups

Participants were divided into BI and NI groups based on a median split of the AMBI score. This methodology was based on previous eyeblink conditioning studies with BI (Caulfield et al., 2013; Holloway et al., 2014) and allowed for equal sample sizes in our BI and NI groups.

### Conditioning session

Upon arrival to the study, participants provided informed consent and were instructed that the study was going to evaluate responses to tones and air puffs to the eye, that they were to watch a silent video of their choice (e.g., a nature video with sound muted), and that they were to remain awake during the testing session. Participants were then fitted with EMG electrodes and headphones, EMG signal quality was verified, and the conditioning program was started. The program began with three US-alone (50 ms, 5.5 psi air puff) exposures to assess UR quality and magnitude for all participants. The acquisition session began immediately following the US exposures. Delay training consisted of 60 acquisition trials and 20 CS-alone extinction trials. The inter-trial interval varied pseudo-randomly between 30 ± 5 s for all contingencies. Participants received either 100% CS-US paired trials or a 50% partial reinforcement schedule for acquisition training. Paired CS-US trials included a 500 ms/1200 Hz pure tone CS overlapping and co-terminating with the US air puff, partial reinforcement schedules included 30 CS-US paired trials inter-mixed with 30 pseudo-random presentations of either a CS alone or US alone trial in which no more than three of the same trial types were presented consecutively.

### Signal processing and data reduction

Electromyography data was evaluated on a trial-by-trial basis for all participants. Processing of eyeblink responses followed methods previously reported (Beck et al., 2008). To determine the occurrence of an eyeblink, EMG activity was first lowpass filtered with a Lowess filter (Stat-Sci, Tacoma, WA, USA) using a time constant of 0.025, and a smoothing interval of 5. With these filter values, activity greater than 0.2 (unitless) corresponded to an eyeblink response. For a response to be counted, smoothed EMG activity in a 500-ms window beginning at the onset of the CS had to exceed the mean activity, plus four times the standard deviation, of the activity in a 125-ms comparator window that immediately preceded the CS window. A CR was scored when an eyeblink occurred 80 ms after CS onset but before US onset. A UR was scored when an eyeblink was produced 0–100 ms after US onset. Those sessions with excessive signal noise (loss of more than 10% of trials), equipment malfunction, or incomplete session data (e.g., falling asleep), were discarded and not used for further analysis. Inspection of the eyeblink conditioning data therefore resulted in rejection of data from 41 participants. The final groups that were analyzed were delay (*n* = 35), 50% partial CS (*n* = 43), and 50% partial US (*n* = 30) for a total of 108.

### Data analysis

To examine the main effects and interactions of anxiety vulnerability and CR acquisition, the 80 trial conditioning session was divided into 10 trial blocks and evaluated independently for 60 acquisition trials and 20 extinction trials. Between group measures included Group (100% CS-US paired trials, 50% CS partial reinforcement, and 50% US partial reinforcement), and BI (BI vs. NI), with Block as a within subject measure. Significant effects from the ANOVAs were followed up with planned F-tests. The planned comparisons included comparisons of BI and NI individuals within each conditioning protocol. In addition, planned comparisons were done between the partial reinforcement schedules with the standard 100% CS-US paired trials condition. The level of significance was set at *p* < 0.05.

## Results

### Psychometric data

Psychometric and demographic data for BI and NI groups for the 100% paired trial, 50% CS partial reinforcement and 50% US partial reinforcement groups are summarized in Table 1. There were no significant gender differences between groups on any of the measures (all *p*’s > 0.13). The AMBI score used for the median split was 11.5 for the 100% paired trials group, 14.5 for the 50% US partial reinforcement group, and 12.5 for the 50% CS Partial reinforcement group.

**Table 1:** **Participant demographics and psychometric data**.

Training protocol	Behavioral inhibition level	*n* (male)	AMBI (se)	Mean %CR acquisition (se)	Mean %CR extinction (se)
100% paired	Non-inhibited	18 (5)	7.7 (0.55)	55.8 (5.4)	33.1 (3.7)
	Inhibited	17 (2)	17.5 (0.80)	59.8 (5.2)	32.1 (5.7)
50% partial CS	Non-inhibited	22 (9)	8.2 (0.60)	45.5 (5.2)	28.2 (4.4)
	Inhibited	21 (18)	19.6 (1.1)	61.0 (4.6)	50.7 (6.0)
50% partial US	Non-inhibited	15 (3)	10.8 (0.73)	46.6 (6.6)	29.3 (5.0)
	Inhibited	15 (4)	19.8 (0.73)	63.6 (5.4)	36.7 (5.1)

### Acquisition

**Figure 1:**
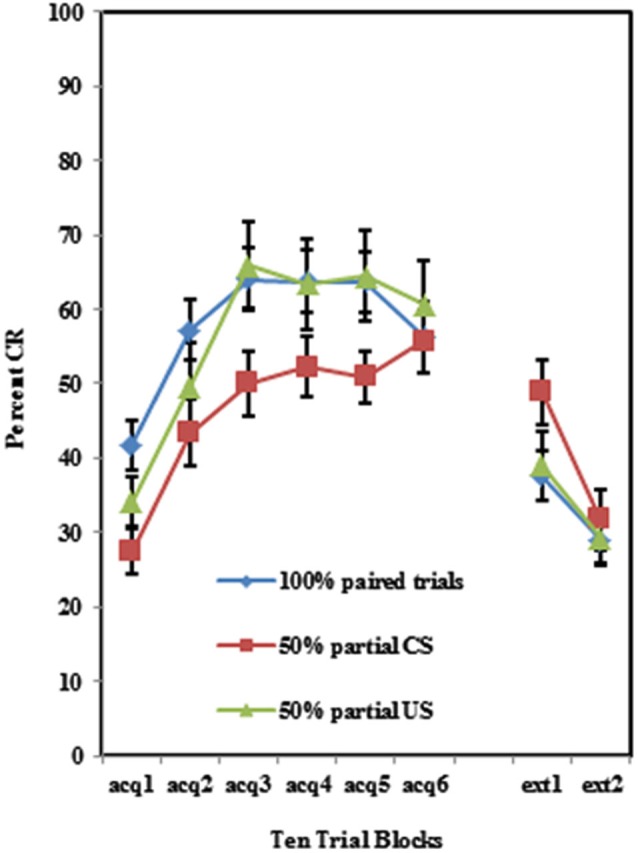
**Percent CRs during 60 acquisition (acq) and 20 CS alone extinction (ext) trials in groups 100% paired trials, 50% partial CS and 50% partial US**. Percent CRs are indicated on the y-axis. The group receiving 50% partial CS trials expressed significantly fewer CRs relative to the 100% paired and 50% partial US groups. Error bars represent standard error of the mean.

Participants acquired CRs across the conditioning session in all three training protocols as shown in Figure 1. This was confirmed with a 3 (Group) × 2 (BI) × 6 (Block) repeated measures ANOVA which revealed a main effect of Block (*F*_(5,510)_ = 29.069, *p* < 0.001). There were significant differences in CR acquisition between the three training protocols. A 3 (Group) × 2 (BI) × 6 (Block) repeated measures ANOVA revealed a main effect of group (*F*_(1,102)_ = 3.226, *p* < 0.05) for conditioned eyeblink response acquisition. Further analysis revealed that the conditioned responding in the 50% CS partial reinforcement protocol was significantly lower than in the 100% paired trial protocol (*F*_(1,74)_ = 6.01, *p* < 0.02). There was no significant group difference between the 100% paired trial protocol and the 50% US partial reinforcement protocol (*p* > 0.70). All interactions for these pairwise comparisons between training protocols were non-significant (*p*’s > 0.25).

**Figure 2:**
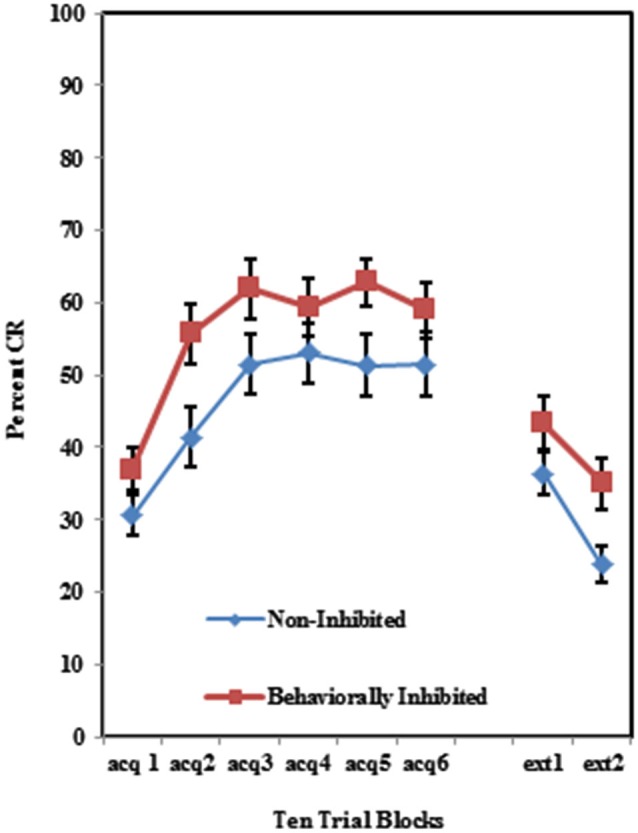
**Percent CRs during 60 acquisition (acq) and 20 CS alone extinction (ext) trials for groups 100% paired trials, 50% partial CS and 50% partial US separated by the median AMBI scores into behaviorally inhibited (BI) and non-inhibited (NI) groups**. Overall, BI individuals expressed significantly more CRs than NI individuals across the 60 trials of acquisition. Behaviorally inhibited individuals also expressed more CRs during the extinction training. Error bars represent standard error of the mean.

As shown in Figure 2, individuals self-reporting high AMBI scores exhibited more CRs across the six acquisition blocks than did those self-reporting low AMBI scores. This was confirmed by a 3 (Group) × 2 (BI) × 6 (Block) repeated measures ANOVA which revealed a significant main effect of BI (*F*_(1,102)_ = 10.596, *p* < 0.005). None of the interactions between these three variables were significant (*p*’s > 0.425).

**Figure 3:**
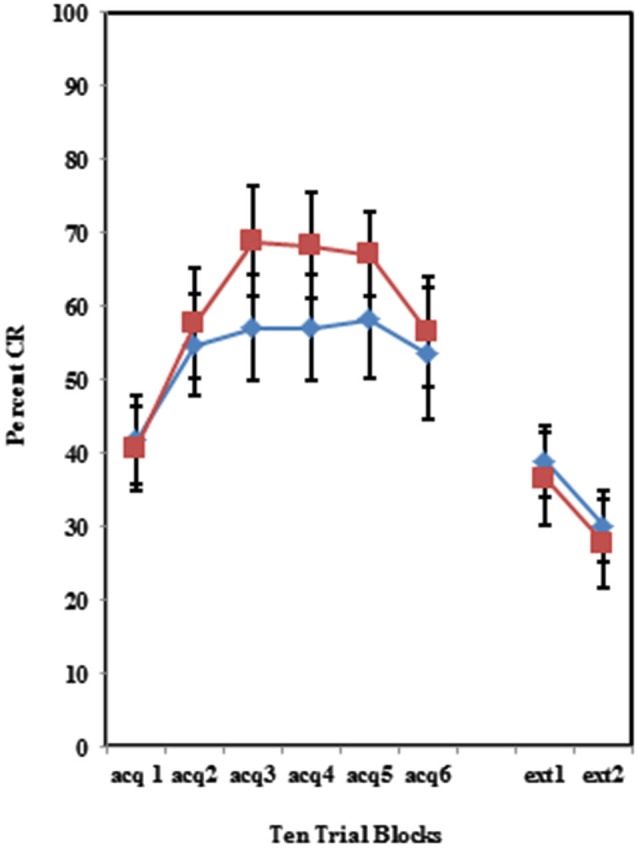
**Percent CRs during 60 acquisition (acq) and 20 CS alone extinction (ext) trials in the group receiving 100% paired trials**. There were no significant differences in acquisition or extinction with 100% paired trials between BI and NI individuals. Error bars represent standard error of the mean.

**Figure 4:**
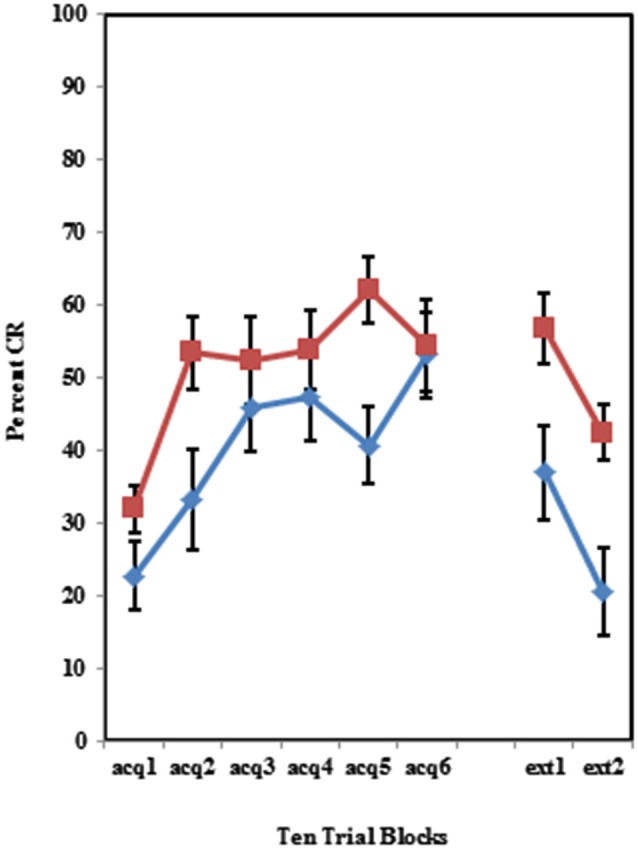
**Percent CRs during 60 acquisition (acq) and 20 CS alone extinction (ext) trials in group receiving 50% partial CS trials**. Behaviorally inhibited individuals expressed significantly more CRs than NI individuals in partial reinforcement training with 50% CS alone trials. Behaviorally inhibited individuals expressed more CRs during the extinction training. Error bars represent standard error of the mean.

**Figure 5:**
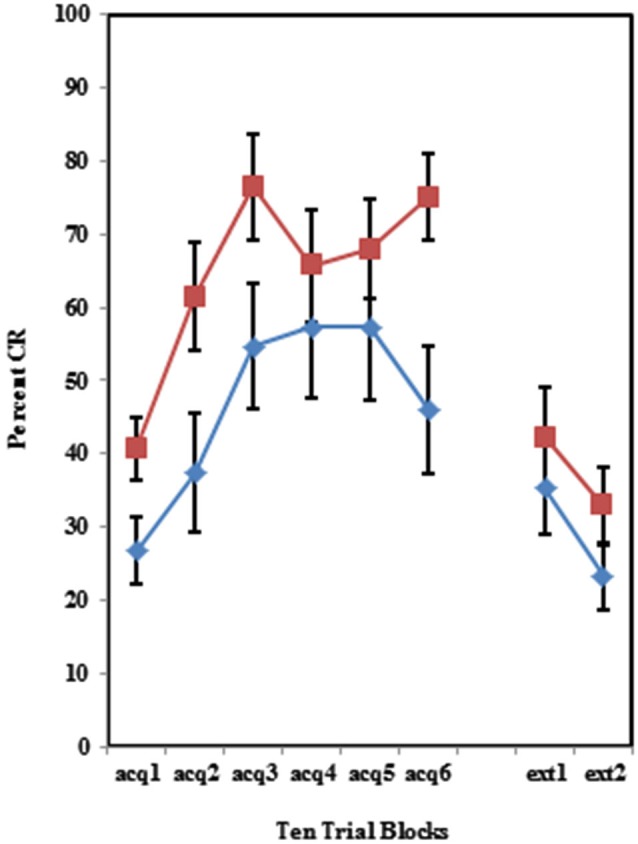
**Percent CRs during 60 acquisition (acq) and 20 CS alone extinction (ext) trials in group receiving 50% partial US trials**. Behaviorally inhibited individuals expressed significantly more CRs than NI individuals in partial reinforcement training with 50% US alone trials, but did not differ during extinction training. Error bars represent standard error of the mean.

The BI effect was further analyzed for each of the individual training protocols separately. Training with the 100% paired trial protocol did not produce a significant difference in conditioned eyeblinks between the high and low AMBI groups as shown in Figure 3. Training with the 50% CS partial reinforcement protocol (*F*_(1,41)_ = 6.469, *p* < 0.05) as well as the 50% US partial reinforcement protocol BI (*F*_(1,28)_ = 4.358, *p* < 0.05) produced significantly more CRs in the high AMBI group as compared to the low AMBI group as shown in Figures 4, 5.

### Extinction

Individuals in all three training protocols exhibited extinction defined by a decrease in conditioned responding across the 20 CS alone trials as evident in Figure 1. This observation was confirmed by a 3 (Group) × 2 (BI) × 2 (Block) repeated measures ANOVA which revealed a main effect of Block (*F*_(1,102)_ = 30.242, *p* < 0.001). Behaviorally inhibited individuals also exhibited more CRs across CS alone trials (i.e., less extinction) than NI individuals as shown in Figure 2. This finding was confirmed by a main effect of BI (*F*_(1,102)_ = 6.263, *p* < 0.05). There was a non-significant trend towards a Group by BI interaction (*F*_(2,102)_ = 3.722, *p* = 0.116).

However, due to significant differences in levels of asymptotic performance across the three training protocols for the high and low AMBI groups, it was necessary to evaluate extinction with respect to the asymptotic performance at the end of acquisition training. The conditioned responding for the last block of acquisition training was used a covariate for further ANOVAs. A 3 (Group) × 2 (BI) ANOVA of these data revealed a significant interaction between Group and BI in conditioned responding during extinction between the three training groups, (*F*_(1,101)_ = 4.25, *p* < 0.05). Based on this interaction, the individual training protocols were evaluated. A significant main effect of BI was evident in the 50% CS alone training protocol (*F*_(1,40)_ = 5.74, *p* < 0.05), but not in the 100% paired trial protocol or the 50% US partial reinforcement protocol (all *p*’s > 0.70).

To analyze for a PREE, pairwise comparisons of the partial reinforcement schedules to the standard 100% CS-US paired trials were conducted. Comparisons of the 50% CS partial reinforcement protocol and the 100% CS-US paired trial protocol revealed a main effect of BI (*F*_(1,73)_ = 5.198, *p* < 0.05) such that BI individuals exhibited more CRs than NI individuals. There was also a significant interaction of Group × BI when the 50% CS partial reinforcement protocol and the 100% CS-US paired trial protocol were compared (*F*_(1,73)_ = 6.272, *p* < 0.05) such there more CRs in the behavioral inhibition group in the 50% CS partial reinforcement condition. There were no significant differences in extinction between the 50% US partial reinforcement schedule and the standard 100% CS-US paired trial protocol.

## Discussion

Prior work by Holloway et al. (2014) found enhanced eyeblink conditioning in individuals self-reporting behavioral inhibition in learning situations such as omission and yoked training in which the pairing of conditioning stimuli was less than optimal. The omission of the US on trials in which a CR was exhibited to the CS resulted in various patterns of partial reinforcement. As conditioning progressed and CRs were acquired, the pairing of the CS and US was reduced. This progressive omission of the US increased participant uncertainty about stimulus pairings. Behaviorally inhibited individuals appeared to be overly sensitive to partial reinforcement schedules in which there is some uncertainty about stimulus pairings and presentations as evidenced by increased conditioned responding as compared to NI individuals.

### Acquisition effects

Partial reinforcement schedules with either 50% CS alone or US alone trials pseudo-randomly inter-mixed with CS-US paired trials produced enhanced acquisition in BI individuals as compared to NI individuals. Our finding of a magnified BI effect in the partial reinforcement schedules with either CS or US alone trials matches the findings of Holloway et al. (2014) with omission and yoked protocols. In addition, our findings with 100% paired trials were similar to those of Holloway et al. (2014) in that while there was a pattern of enhanced acquisition BI in the 100% paired trials, this difference was not significant. It appears both in our present work with partial reinforcement schedules and prior work with omission and yoked controls that the effects of BI are most evident in non-optimal conditions in which CS-US pairings are less than 100%.

One feature of BI is an enhanced reactivity to novelty, threat, and uncertainty (Hirshfeld et al., 1992; Schwartz et al., 2003a,b). The current partial reinforcement protocols with either CS or US alone presentations pseudo-randomly intermixed with CS-US paired trials produced uncertainty for both stimulus presentation and timing of CS-US paired trials for the participants. During the training session, it is not apparent to the participant when the next CS-US paired trial will occur. In the case of the CS alone partial reinforcement protocol, the next trial could either be a CS paired with a US and should be responded to or it could be a CS without an US and does not need to be responded to. In the case of the US alone partial reinforcement protocol, the next trial could either be a CS paired with a US which should be responded to or be an un-signaled US. This uncertainty was also found with the yoked group in Holloway et al. (2014) when the participants received what appeared to be a random arrangement of CS-US paired trials and CS alone trials based on the CR performance of their matched omission participant. The current findings support the further exploration of uncertainty as an important feature of enhanced learning in BI individuals.

In addition to our findings with BI, the schedules of partial reinforcement differed from the standard 100% CS-US paired training. Partial reinforcement training with CS alone trials was found to produce less conditioned responding than 100% paired CS-US training. This finding corresponds to prior human eyeblink conditioning studies with partial reinforcement with CS alone trials (Reynolds, 1958; Ross, 1959; Hartman and Grant, 1960; Ross and Spence, 1960; Runquist, 1963; Perry and Moore, 1965).

In contrast to the findings with CS alone trials, eyeblink conditioning with the 50% US partial reinforcement protocol did not differ from 100% paired CS-US trials. This finding was somewhat surprising in that only half of the trials were training trials (i.e, CS-US pairings). However, the presentation of a corneal air puff alone could be a viewed as unexpected. The unexpected nature of these US alone trials could facilitate conditioning through several neural mechanisms involving attention.

Several theories have proposed the reinforcement system for different forms of motor learning in the cerebellum (including classical eyeblink conditioning) to be the climbing fiber system from the inferior olive (Albus, 1971; Eccles, 1977; Ito, 1982; Thompson, 1989; Swaim et al., 2011). The inferior olive climbing fiber system has been hypothesized as a teaching signal for the cerebellum. This cerebellar circuitry has been hypothesized by several theories and computational models to work in an error correction manner similar to the Rescorla-Wagner rule (e.g., Kenyon et al., 1998; Gluck et al., 2001). In these models, the error correction between the actual US and a prediction of the US (i.e., the CR) is instantiated as an inhibitory connection between the cerebellum and the inferior olive. The enhanced conditioning in the case of partial reinforcement training with US alone trials may be due to this circuit. Sears and Steinmetz (1991) found that inferior olive activity is inhibited on CR trials but is present on trials in which a CR does not occur. This pattern of US firing during US alone presentations intermixed with CS-US paired trials may produce the higher numbers of CRs in the partial reinforcement protocol with US alone trial. The random inferior activity could “spark” plasticity in the cerebellum leading to more CRs than would be expected with only 50% CS-US paired trials.

Another way in which attentional mechanisms may modulate the cerebellum is via theta activity from the septo-hippocampal system. Theta activity enhances eyeblink conditioning in rabbits (Berry and Seager, 2001) while disruption of the septo-hippocampal system via medial septal lesions or administration of cholinergic antagonists slows delay eyeblink conditioning (Berry and Thompson, 1979; Allen et al., 2002). Gray and McNaughton (2000) proposed theta is also associated with anxiety in that the septo-hippocampal system responds to competing options or motivations (possibly due to uncertainty) by increasing vigilance. Theta activity, thus, may be a source of the enhanced acquisition observed in BI individual in conditioning situations where there is less than optimal relationships between stimuli.

### Extinction effects

In addition to findings of BI and partial reinforcement effects on acquisition of conditioned eyeblinks, the current study also revealed differences in extinction to CS alone presentations for BI individuals. Previous work with omission and yoked protocols (Holloway et al., 2014) did not produce any effects of BI on extinction even though about 50% of the trials were CS alone due to the omission of the US air puff on CR trials. In the present study, BI individuals exhibited a partial reinforcement extinction effect (i.e., PREE) following CS alone partial reinforcement such that they responded more during CS alone extinction trials as compared to individuals trained with 100% CS-US paired trials. However, NI individuals did not show PREE: i.e., those trained with CS alone partial reinforcement did not differ in extinction from those trained with 100% CS-US trials. A subset of prior classical conditioning studies with partial reinforcement with CS alone trials have reported a PREE (Longenecker et al., 1952; Perry and Moore, 1965; Newman, 1967; Leonard, 1975). Based on the current findings, the inconsistency in the past in obtaining a PREE in human eyeblink conditioning could be explained by temperament factors such as behavioral inhibition. It is of interest to note that while there is a large body of anxiety work and partial reinforcement studies with eyeblink conditioning from the 1950’s and 1960’s, the current study is the first to combine both elements.

Some aspects of the BI effect on extinction can be explained through the hippocampal modulation of eyeblink conditioning. Hippocampal lesions have been found to disrupt extinction of conditioned eyeblinks to tone alone training in rabbits (Schmaltz and Theios, 1972; Akase et al., 1989). Hippocampal theta activity also plays a role in the PREE. Gray (1972) found that theta is highest on non-reward trials and that medial septal lesions or electrical stimulation that blocks theta activity also disrupts PREE in rat straight alley maze running for a water reward.

Additionally, Penick and Solomon (1991) found that hippocampus is involved in encoding context. The hippocampal encoding of context may be responsible for the differences in extinction between 50% CS partial and the 100% CS-US paired protocols found with BI individuals. Spence et al. (1964) found an inverse relationship between the rate of extinction and recognition of changes in trial type between acquisition and extinction training. In the case of our CS alone partial reinforcement protocol, the extinction phase was similar to the acquisition phase in that both included CS alone presentations. This similarity in context may have contributed to the continued conditioned responding to the CS alone trials in high BI individuals. The PREE observed with CS alone training could be interpreted as being due to consistencies in context between acquisition and extinction training. Differences in extinction between BI and NI may be due to differential hippocampal activity based on continued vigilance to the CS alone presentations.

### Limitations and conclusions

The sample for the current study had a few limitations. First, the participants were undergraduates in psychology courses who voluntarily participated for research credit for coursework. While it is possible the participants had some preconceptions about the nature of the study, they were blind to the fact that they were going to do eyeblink conditioning and were also blind to the type of training protocol with which they would be presented.

Second, the sample included a majority of female participants (i.e., 98 females as compared to 51 males). While anxiety disorders are more prevalent in females, and females have also been reported as exhibiting facilitated eyeblink conditioning (Spence and Spence, 1966), the present study did not observe a gender effect for eyeblink acquisition which matches with other recent eyeblink conditioning studies concerning BI (Caulfield et al., 2013; Holloway et al., 2014). There were also no significant differences between males and females for any of the demographic measures. Third, the present study utilized a non-clinical population of college undergraduates who self-reported anxiety vulnerability on the AMBI scale. One unanswered question is whether the current findings would generalize to a post-traumatic stress disorder (PTSD) population or other anxiety disorder populations. Myers et al. (2011) found enhanced eyeblink conditioning in a delay paradigm with 100% paired trials among veterans self-reporting severe PTSD symptoms. It would be of interest to test the current findings of even greater enhancement of eyeblink conditioning in the partial reinforcement conditions in a population that has been clinically diagnosed with PTSD or some other anxiety disorder. The pattern of faster acquisition and slower extinction in partial reinforcement is similar to the symptoms of PTSD.

Our working hypothesis was that temperament factors like BI may alter associative learning thus leading to increased risk of development of anxiety disorders when presented with aversive stimuli. The current findings with partial reinforcement protocols match previous findings with omission and yoked protocols (Holloway et al., 2014). Behaviorally inhibited individuals exhibited greater facilitation of eyeblink conditioning (i.e., associative learning) at a greater rate in partial reinforcement protocols than in standard 100% paired trials. Additionally, the partial reinforcement protocol with CS alone trials revealed a PREE effect in only the high BI individuals. The current study furthers our understanding of enhanced associative learning in individuals self-reporting behavioral inhibition. Overall, this work supports a growing literature in which enhanced associative learning, especially in the cases where there is some uncertainty, is an elemental component of anxiety disorders.

## Conflict of interest statement

The authors declare that the research was conducted in the absence of any commercial or financial relationships that could be construed as a potential conflict of interest.
